# S14-2: The association of physical activity referral scheme’ components with physical activity level, scheme uptake and adherence rates: a systematic review, meta-analysis with meta-regression

**DOI:** 10.1093/eurpub/ckae114.261

**Published:** 2024-09-26

**Authors:** Wolfgang Geidl, Anna Brandmeier, Sarah Klamroth, Inga Naber, Anja Weissenfels, Karim Abu-Omar, Klaus Pfeifer, Eriselda Mino, Coral Hanson, Sheona McHale, Michael Schuler, Peter Gelius, Stephen Whiting, Kremlin Wickramasinghe, Gauden Galea

**Affiliations:** Friedrich-Alexander University Erlangen-Nürnberg, Department of Sport Science and Sport; Friedrich-Alexander University Erlangen-Nürnberg, Department of Sport Science and Sport; Friedrich-Alexander University Erlangen-Nürnberg, Department of Sport Science and Sport; Friedrich-Alexander University Erlangen-Nürnberg, Department of Sport Science and Sport; Friedrich-Alexander University Erlangen-Nürnberg, Department of Sport Science and Sport; Friedrich-Alexander University Erlangen-Nürnberg, Department of Sport Science and Sport; Friedrich-Alexander University Erlangen-Nürnberg, Department of Sport Science and Sport; Friedrich-Alexander University Erlangen-Nürnberg, Department of Sport Science and Sport; Edinburgh Napier University UK, School of Health and Social Care; Edinburgh Napier University UK, School of Health and Social Care; University of Würzburg, Institute of Clinical Epidemiology and Biometry; Université de Lausanne Switzerland, Institute of sport sciences; WHO Regional Office for Europe Copenhagen Denmark, Special Initiative for Noncommunicable Diseases and Innovation (SNI); WHO Regional Office for Europe Copenhagen Denmark, Special Initiative for Noncommunicable Diseases and Innovation (SNI); WHO Regional Office for Europe Copenhagen Denmark, Special Initiative for Noncommunicable Diseases and Innovation (SNI)

## Abstract

**Purpose:**

Physical activity referral schemes (PARS) are complex interventions comprised of multiple components such as screening, brief advice, and written prescription. This study aimed to investigate the relationship between PARS components and physical activity, uptake, and adherence rates. Additionally, the overall effect of PARS is analysed.

**Methods:**

6 databases were searched for studies published between 1990-2023; included PARS with participants ≥16 years old; and reported either physical activity, uptake or adherence outcomes. GRADE was used to assess quality of evidence. Separate random-effects meta-analysis by comparison group were conducted for physical activity. Uptake and adherence rates were pooled using proportional meta-analysis. The PARS components were analyzed via univariate meta-regression.

**Results:**

We included 52 studies from which 50 targeted people with, or at risk of, non-communicable diseases. PARS were compared with usual care (11 RCTs, Hedges’ g = 0.18, 95%CI 0.12 to 0.25), physical activity advice (5 RCTs, Hedges’ g=-0.6, 95%CI -0.21 to 0.10), or enhanced PARS (9 RCTs, Hedges’ g = 0.07, 95%CI -0.03 to 0.18). The pooled uptake rate was 87% (95%CI 77% to 94%) among 14 RCTs and 68% (95%CI 51% to 83%) among 14 non-experimental studies. The adherence rate across 16 RCTs and 18 non-experimental studies was 68% (95%CI 55% to 80%) and 53% (95%CI 42% to 63%) respectively.

The meta-regression showed that PARS incorporating a person-centered approach, screening, and brief advice had higher adherence rates. In contrast, PARS offering physical activity sessions had lower adherence rates. No other component-outcome relationship reached statistical significance.

**Conclusion:**

High certainty of evidence confirms a small effect of PARS in increasing physical activity compared to usual care. The evidence comparing PARS with advice and enhance scheme versions is inconclusive and comes from low certainty of evidence. Four out of the 19 PARS components may predict adherence.

**Practical implications:**

The results improve our understanding of effective PARS components in relation to physical activity promotion, scheme uptake and adherence and thus support the future development of optimal PARS.

**Funding:**

The study was conducted within the project BewegtVersorgt, which is funded by the Federal Ministry of Health based on a resolution of the German ‘Bundestag’ by the federal government.

